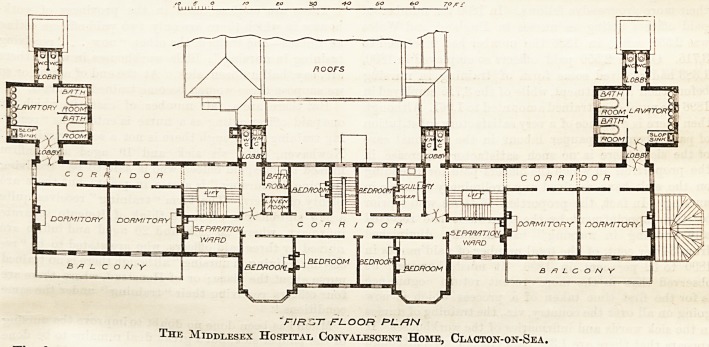# Hospital Construction

**Published:** 1897-05-08

**Authors:** 


### THE INSTITUTIONAL WORKSHOP.

### HOSPITAL CONSTRUCTION.

### THE MIDDLESEX HOSPITAL CONVALESCENT HOME, CLACTON-ON-SEA.

The site of the new home erected by the Weekly Board of the Middlesex Hospital is 5 acres in extent and lies about 400 yards back from the edge of the cliff. In form it is a triangle with its base on the south and towards the sea. The building has been planned to accommodate some 56 patients (men, women, and children), with room for four sick nurses—60 in all, and with a view to possible future extension.

THE MIDDLESEX HOSPITAL CONVALESCENT HOME
CLACTON-ON-SEA

GROUND FLOOR PLAN

The main front building is three storeys in height and faces almost due south. The central part contains the rooms for the staff and the sick nurses' rooms, the east wing being devoted to male patients and the west wing to women and children.

On the ground floor is the entrance hall, committee room, matron's sitting-room, and sitting-room for nurses; all these face south. At the back is a large linen room, a box room, and a bedroom intended for the use of the resident medical officer or other member of the staff when necessary. A staircase in the central part affords separate access to the rooms for the staff on the upper floor.

Each wing contains on the ground floor two large day-rooms and a surgery. The provision of two surgeries is an unusual one, but is of great practical convenience. It keeps the patients to their own part of the house, and helps discipline. On the male side is a wood and glass smoking-room. In the well-hole of each staircase is a hand-lift for coals, linen, &c.

with nurses' room and small pantry, is a separate block, connected with the main building and the kitchen block by open covered ways.

North of the main group of buildings, and connected by a covered way, is the laundry block and the boiler and engine house. The latter is fitted with a complete installation of electric machinery in duplicate for producing light for all the buildings, and for providing motive power for the laundry.

At one end of the boiler house is the water tower. The water, which is obtained from a well on the site, is pumped to the top of the tower, where it undergoes a process of softening (Tyacke patents), and is then pumped to tanks in the roof of the main building.

Provision is made in the building for a disinfecting apparatus and an ambulance, neither of which have yet been obtained.

The upper floors of the front building contain the dormitories for patients with the bed-rooms for staff. On each floor and on each side is a separation ward for one bed.

THE MIDDLESEX HOSPITAL CONVALESCENT HOME, CLACTON-ON-SEA.

The lavatories are placed in projecting wings connected with the main building by cross-ventilated corridors. On the ground floor the room marked "lavatory" on the plan is really a combined cloak and boot room and lavatory. Here each patient is provided with a bin for his or her boots, a hook for coat or cloak, and one for towel. The lavatory basins are made each in a solid piece of fireclay built into the wall, so that no support or bracket is needed.

On this floor the water closets are separated by open lobbies, which form also the means of egress and ingress for the patients.

The dining hall is placed at the north of the main building, and access is gained to it from each side for male and female patients respectively. The kitchen and scullery are at the back of the dining hall, with a small serving-room provided with a steam-heated carving-table between the hall and the kitchen. On the west side of the passage leading from the female side to the kitchen offices are the nurses' dining-room, servants' hall, with pantry between, tradesmen's entrance, stores, larder, and boot-room. The isolation ward,

The building has been constructed of brick, with weather tiling on the upper floors, and a small part of half-timber work. On the ground floor the day-rooms open on to broad verandahs, which give access to the garden; on the first floor the flat roofs of these verandahs become balconies of sufficient width to admit of a bed being wheeled out.

The corridors and staircases throughout are of cement-concrete, finished with Walker's patent paving. All corridors, the lavatories, and the dining-hall are warmed by hot-water radiators.

In addition to the main building, there is a lodge at the front entrance and one at the north end of the site; also a greenhouse, with potting-shed and seed-room, and a set of piggeries.

The building was designed by Mr. Keith D. Young, F.R.I.B.A., architect to the hospital, in consultation with Mr. E. A. Fardon, the resident medical officer. Miss Smedley, matron of the Children's Hospital, Great Ormond Street, and formerly matron of the Hospital's Convalescent Home, Swanley, materially helped, when the plans were being worked out, by many practical suggestions.

## Figures and Tables

**Figure f1:**
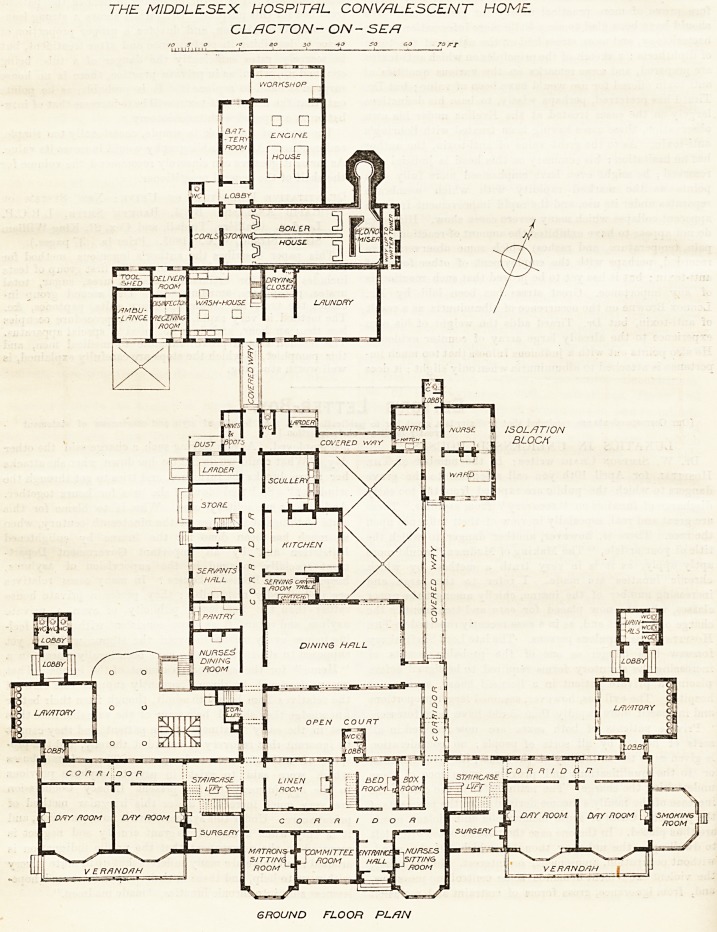


**Figure f2:**